# A Retrospective Analysis of the Correlation between Functional Imaging and Clinical Outcomes in Grade 3 Neuroendocrine Tumors (NETs G3)

**DOI:** 10.3390/diagnostics11122401

**Published:** 2021-12-20

**Authors:** Alice Laffi, Marzia Colandrea, Giuseppe Buonsanti, Samuele Frassoni, Vincenzo Bagnardi, Francesca Spada, Eleonora Pisa, Massimo Barberis, Manila Rubino, Chiara Maria Grana, Francesco Ceci, Nicola Fazio

**Affiliations:** 1 Division of Gastrointestinal Medical Oncology and Neuroendocrine Tumors, European Institute of Oncology (IEO), IRCCS, Via Ripamonti 435, 20141 Milan, Italy; francesca.spada@ieo.it (F.S.); manila.rubino@ieo.it (M.R.); nicola.fazio@ieo.it (N.F.); 2 Division of Nuclear Medicine, European Institute of Oncology (IEO), IRCCS, Via Ripamonti 435, 20141 Milan, Italy; marzia.colandrea@ieo.it (M.C.); segreteria.medicinanucleare@ieo.it (G.B.); Francesco.ceci@ieo.it (F.C.); 3 Department of Statistics and Quantitative Methods, University of Milano-Bicocca, 20126 Milan, Italy; samuele.frassoni@unimib.it (S.F.); vincenzo.bagnardi@ieo.it (V.B.); 4 Division of Pathology and Laboratory Medicine, European Institute of Oncology (IEO), IRCCS, Via Ripamonti 435, 20141 Milan, Italy; ufficio.apa@ieo.it (E.P.); massimo.barberis@ieo.it (M.B.); 5 Unit of Radiometabolic Therapy, European Institute of Oncology (IEO), IRCCS, Via Ripamonti 435, 20141 Milan, Italy; chiara.grana@ieo.it

**Keywords:** NET G3, neuroendocrine, PET/CT, ^68^Ga, ^18^F-FDG

## Abstract

Grade 3 (G3) neuroendocrine tumors (NETs) are a novel category among digestive neuroendocrine neoplasms, characterized by Ki-67 >20% and a well-differentiated morphology, presenting high intra-tumor heterogeneity. We aimed to explore the role of dual-tracer PET imaging (^68^Gallium (Ga)-DOTATOC and ^18^Fluorodeoxyglucose (FDG)) as overall survival (OS) predictor in NET G3 patients. We performed a retrospective analysis in NET G3 patients treated at our institution between 2003 and 2021. Accordingly, 30 NET G3 patients were analyzed. ^68^Ga-DOTA-TOC and ^18^F-FDG uptake were assessed by tumor/non-tumor (T-nonT) ratio. We reported a slightly better OS for patients with ≥75% concordance between ^68^Ga-DOTA-TOC and ^18^F-FDG PET/CT (*p* = 0.42). Among patients with discordant functional imaging, we reported a better 5-y OS rate for patients with a prevalent ^68^Ga-DOTATOC vs. ^18^F-FDG PET/CT (*p* = 0.016). In positive ^18^F-FDG PET/CT cases, we reported a better OS for <4 vs. ≥4 T/non-T ratio (*p* = 0.021). Among upfront-NET G3 patients with concordant exams, 5-y OS rate was 83.3% (95% CI: 27.3–97.5). Among patients with discordant exams, 5-y OS rate was 81.3% (52.5–93.5), 100% for those with prevalent receptor expression, and 50% (11.1–80.4) for those with prevalent ^18^F-FDG uptake. Our findings suggest that dual-tracer PET/CT can be considered as a predictor of patient outcome, able to stratify NET G3 patients with poorer prognosis.

## 1. Introduction

Gastro-entero-pancreatic (GEP) neuroendocrine tumors (NETs) represent a category of rare and well-differentiated neoplasms, well-known for a more indolent biologic behavior compared with their non-neuroendocrine counterparts [1,2]. Previously, high-grade neuroendocrine neoplasms (NENs) comprised poorly and well-differentiated NENs under the name of “neuroendocrine carcinomas” (NECs). Later, the World Health Organization (WHO) classification of GEP NENs introduced the NET G3 category, a novel entity, morphologically and prognostically separated from poorly-differentiated NECs [3,4]. The diagnosis of NET G3 is based on two pathologic features: a high proliferation index (Ki-67 >20%), and a well-differentiated morphology. Although several authors argue that NETs G3 Ki-67 usually range between 20 and 50% [5], the present classification of GEP NENs does not specify the upper limit. Therefore, NETs G3 might virtually present a proliferation index from 20% to 100% as well as maintaining a well-differentiated morphology. Since its introduction, different clinical, pathological, and radiological (including positron emission tomography, PET) features within the category suggested a greater heterogeneity than presumed, and the need to develop a multiparametric system of the NET G3 classification has grown. As recently observed in a retrospective analysis performed by our research group in NET G3 patients in these three different settings, we found some interesting prognostic factors according to the timing of developing a NET G3 (if NET G3 was the first diagnosis of NET for a patient or if it occurred during a history of a previous G1/2 NET), the proliferation index, and ^68^Gallium and FDG uptake [6]. Therefore, this study was designed to explore the role of dual-tracer PET imaging (both using ^18^F-FDG and ^68^Ga-DOTA-TOC) in NET G3 patients and to assess possible associations with clinical outcomes.

## 2. Materials and Methods

### 2.1. Study Design and Patient Population

We retrospectively reviewed the clinical records of NEN patients from January 2003 to January 2021, evaluated by a single-center (IEO, European Institute of Oncology), multidisciplinary tumor board center of excellence for GEP NETs certified by the European Neuroendocrine Tumor Society—ENETS. 

Clinical and pathological records were reviewed excluding all duplicates and all reports referring to patients with poorly-differentiated NECs, NETs from extra-GEP sites, or NET G1-2. Patients with lost follow-up were discarded by this analysis. We selected only well-differentiated NENs with a Ki-67 >20% (NET G3) from GEP and from an unknown primary site strongly suspected to be of gastrointestinal origin (immunohistochemical features, metastatic sites, and/or clinical syndrome). All selected cases were diagnosed or reviewed by three NEN-referral pathologists in accordance with the 2019 WHO diagnostic criteria for GEP NENs. 

According to the literature, the whole population has been stratified according to clinical and radiological features. We used the term “late NET G3” to define patients with a NET G3 diagnosis that occurred over G1/2 NET clinical history and “upfront NET G3”, those with a NET G3 diagnosis coinciding with the NEN diagnosis.

The study was approved by our local internal review board (IRB) and the Data Protection Officer at our institute (U-ID: 2546). Every patient gave their consent for the research, as required by the institution regulation for retrospective analyses. All collected data were anonymized. 

### 2.2. Radiological Selection and Characterization

We collected functional imaging performed at the time of diagnosis for all upfront-NET G3 patients; for late-NETs G3, we collected functional imaging performed at the time of G1/G2 NET diagnosis and those at the time of the later NET G3 diagnosis. We excluded all the cases lacking at least one (receptorial or metabolic) functional imaging. A central imaging review of all selected cases was performed by a NEN-dedicated team of nuclear medicine specialists.

In accordance with the distribution of receptorial and metabolic radiotracers, PET/CT image interpretation has been in the “homogeneous” and “inhomogeneous” sub-categories according to visual criteria. Referring to the Krenning scale (K-score) for receptorial expression [7] and Deauville criteria (DC) for metabolic radiotracer uptake [8], the interpretation of each radiopharmaceuticals was grouped as: low uptake, when it was equal to or below blood pool—mediastinum (grade 1 K-score and point 2 DC), moderate uptake, when the uptake was above mediastinal, but below or equal to uptake in the liver (grade 2 K-score and point 3 DC), and high uptake when it was higher than the liver uptake (grade 3–4 K-score and point 4–5 DC). The lack of receptors or uptake corresponded to negative exam (which meant point 1 DC, grade 0 K-score).

Therefore, ^68^Ga-DOTA-TOC-PET/CT was defined as homogeneous when the lesions had a high and uniform distribution of the radiotracer. Somatostatin analogue (SSA) therapy was suspended at least 15 days before the exam. In turn, ^68^Ga-DOTA-TOC PET/CT was defined as inhomogeneous when not all lesions had a high and uniform distribution of the radiotracer among them. In these cases, there may be lesions lacking receptorial expression or with a low/moderate distribution of the radiotracer, besides others with a high distribution of the radiotracer. We termed homogeneous ^18^F-FDG-PET/CT where there was a high and uniform ^18^F-FDG uptake among the lesions. In turn, we defined inhomogeneous ^18^F-FDG-PET/CT where there was a different radiotracer uptake (1) between the primitive tumor and metastases, or (2) among metastases (at least one target lesion with a different uptake). We defined as “concordant” the results of ^68^Ga-DOTA-TOC PET/CT and ^18^F-FDG-PET/CT when the radiotracers reported the same grade of distribution in at least 75% of the lesions among the two exams. In turn, the results of functional imaging were defined as “discordant” when (1) ^68^Gallium-DOTA-TOC-PET/CT was negative and ^18^F-FDG-PET/CT positive or (2) vice versa, and (3) when the radiotracers reported the same grade of distribution in <75% of the lesions among the two exams. When the two functional imaging results were discordant, we specified the most prevalent exam, meaning the one detecting the greatest part of the tumoral mass. For late-NET G3, we also analyzed the concordance/discordance between dual-tracers PET/CT performed before and after the NET G3 diagnosis. Finally, in cases of positive ^18^F-FDG PET/CT, we analyzed the tumor/non-tumor ratio (T-nonT), which means the ratio between the highest standardized uptake value (SUVmax) in tumor lesions and mediastinal blood pool SUVmax, setting a region of interest (ROI) with a diameter of 15 mm. 

### 2.3. Statistical Analysis

Continuous variables were reported as median and ranges, while categorical variables, as counts and percentages, were reported overall and stratified by the two subgroups as upfront and late NET G3. Wilcoxon’s signed-rank test for continuous variables and Fisher’s exact test for discrete variables were used to compare the distribution of the evaluated characteristics between groups. The OS was estimated from the date of G3 diagnosis to death or last contact, using the Kaplan–Meier method. The log-rank test was performed to evaluate differences between groups. All analyses were performed with the statistical software SAS 9.4 (SAS Institute, Cary, NC, USA).

## 3. Results

**Figure 1:**
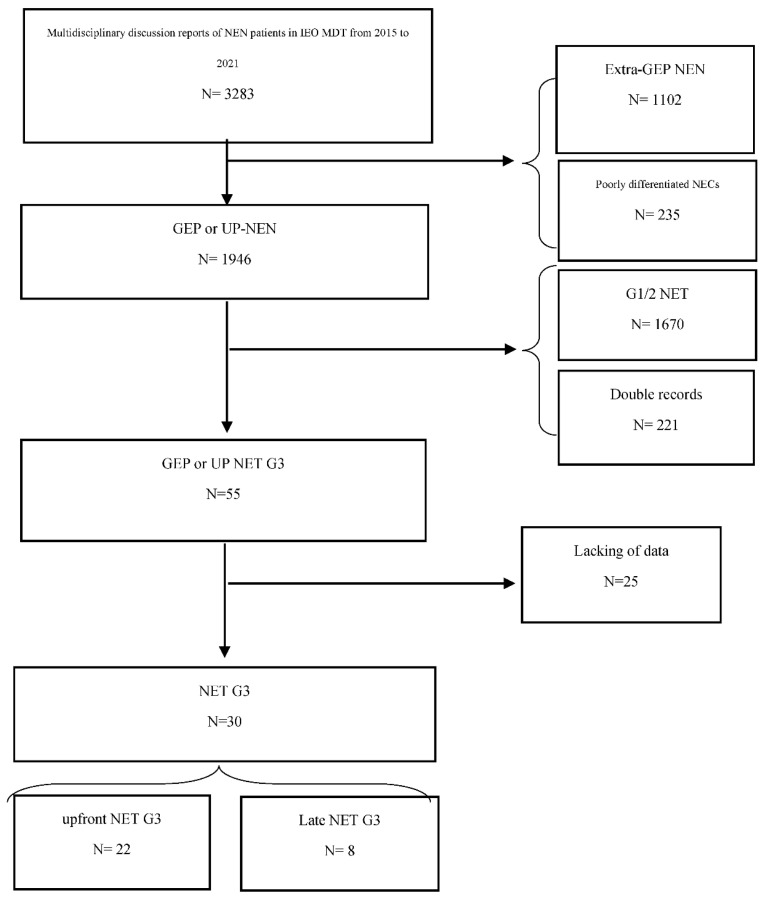
CONSORT flow chart.

According to the CONSORT flow chart reported in Figure 1, we selected 30 patients from the reports of the NEN-dedicated multidisciplinary team (MDT): 22 with upfront-NET G3 and eight with late-NET G3. In two patients, the primary site was not identified; however, immunohistochemical features, clinical syndrome, and metastatic sites were strongly suggestive of an intestinal origin. The main clinical and radiological features of the sample was reported in Table 1.

According to the inclusion criteria, all patients underwent receptorial and metabolic PET/CT imaging. The majority of the receptorial and metabolic PET/CT were performed simultaneously (median time 0 mo, range 0–24) and all but one within 3 mo. For patients with late-NET G3, the median time between dual-tracer PET after NET G3 diagnosis was 1.5 mo (0–8). Within upfront-NET G3, ^68^Ga-DOTA-TOC and ^18^F-FDG-PET/CT were negative in two and five patients respectively, while no patients had negative functional imaging among the late-NET G3. In both upfront and late-NET G3, ^68^Ga-DOTA-TOC PET/CT images were prevalently homogenous vs. inhomogeneous, while ^18^F-FDG-PET/CT was equally distributed between homogeneous and inhomogeneous in both subgroups. 

The majority of patients (21/30, 70%) presented a discordance between ^68^Ga-DOTA-TOC and ^18^F-FDG PET/CT, mostly among upfront-NET G3. Fifteen out of 21 patients (71%) showed a prevalence of ^68^Ga-DOTA-TOC on the ^18^F-FDG PET/CT, mostly within upfront-NET G3. Only nine patients reported a concordance of ≥75%, eight a complete concordance between functional imaging. Among patients with discordant functional imaging, 10 had completely or almost completely discordant functional imaging (0–25% of concordance): 9/10 showed a homogeneous ^68^Ga-DOTA-TOC PET/CT on all lesions and an FDG uptake in only few metastases, while 1/9 showed a homogeneous ^18^F-FDG PET/CT and a negative ^68^Ga-DOTA-TOC PET/CT. Finally, among patients with positive ^18^F-FDG PET/CT, we reported an equal distribution of < vs. ≥4 T/non-T between upfront and late-NET G3 subgroups.

The median follow-up was 24 months (2–146) with a 5-y OS of 83.3% (64.5–92.7). No significant differences were reported between upfront vs. late-NET G3, by gender or age (< or ≥50 y), while pancreatic NET G3 showed a small trend to a worse OS vs. other gastrointestinal origin sites (5-y OS 80% vs. 90%). A significant 5y-OS was reported for ≤ vs. >30% Ki-67 NET G3 patients (*p* = 0.049). 

**Figure 2:**
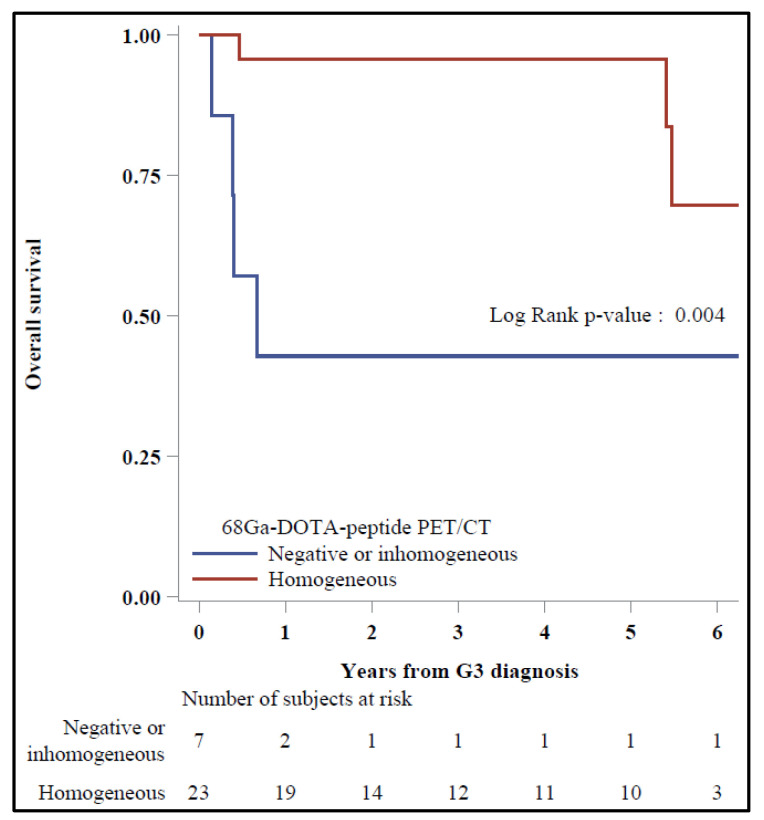
Overall survival by ^68^Ga-DOTA-DOT PET/CT (*N* = 30).

**Figure 3:**
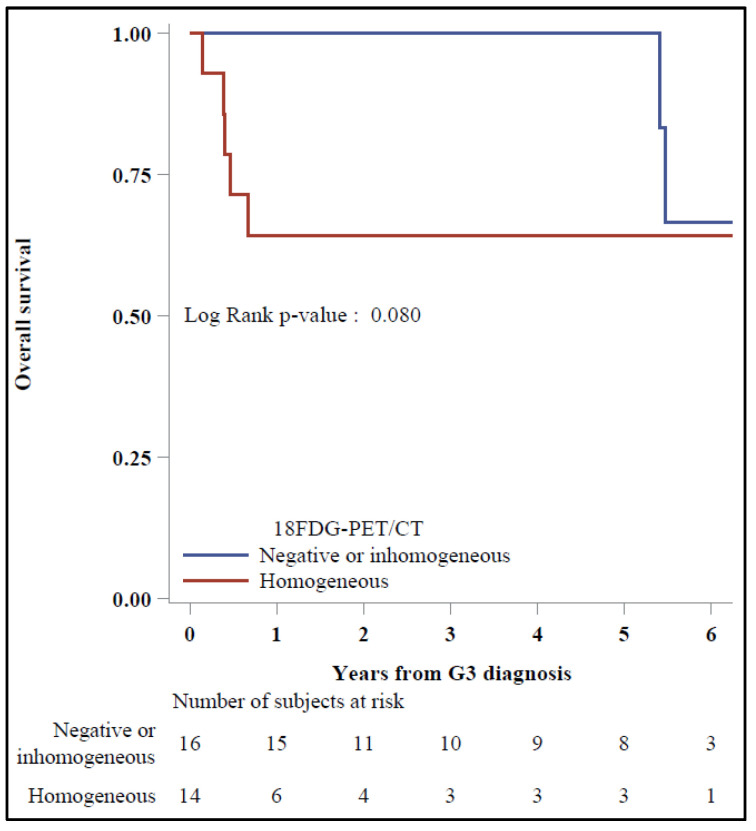
Overall survival by ^18^F-FDG-PET/CT (*N* = 30).

In the Kaplan–Meier plots, a significant 5-y OS was reported for patients with homogeneous vs. negative/inhomogeneous ^68^Ga-DOTA-TOC PET/CT (95.7% vs. 42.9%, *p* = 0.004) (Figure 2), while 5-y OS was 100% vs. 64.3%, respectively, for those with negative/inhomogeneous vs. homogeneous ^18^F-FDG PET/CT (*p* = 0.08) (Figure 3). 

**Figure 4:**
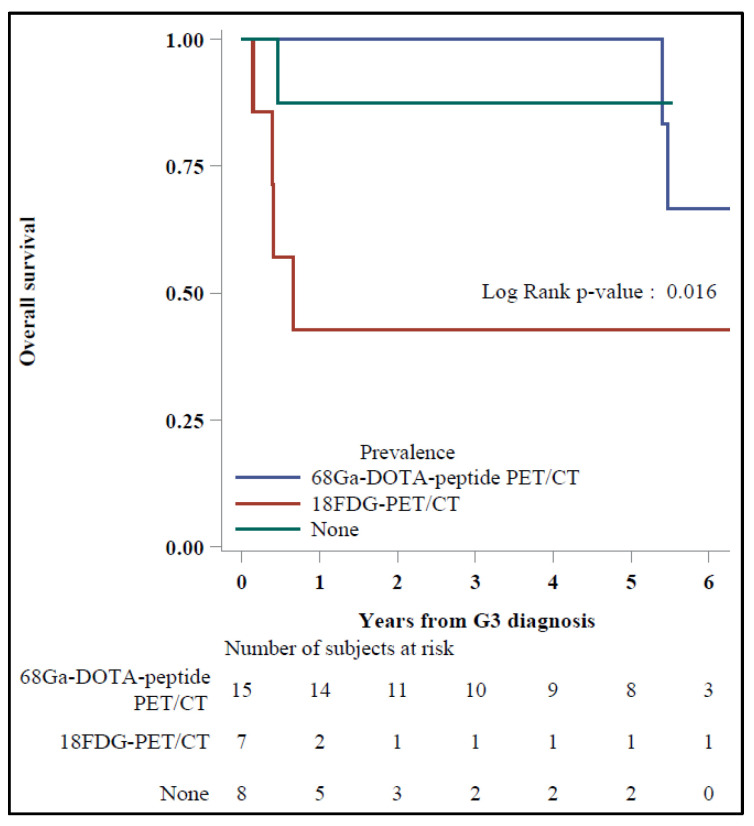
Overall survival by prevalence.

A trend toward a better OS was also reported for patients with a concordance ≥75% between ^68^Ga-DOTA-TOC and ^18^F-FDG PET/CT vs. the others with discordant functional imaging; regarding the prevalent exam, the 5-y OS was 100% vs. 42.9%, respectively, for patients with prevalent ^68^Ga-DOTA-TOC vs. ^18^F-FDG PET/CT (*p* = 0.016) (Figure 4). 

Among the upfront-NET G3, we reported six and 16 patients with concordant and discordant exams, respectively. The 5-y OS of the first was 83.3% (27.3–97.5); one had died at the time of the analysis. The 5-y OS of the second was 81.3% (52.5–93.5), 100% for those with a prevalent ^68^Ga-DOTA-TOC-PET/CT (all alive at the time of the analysis) and 50% (11.1–80.4) for those with a prevalent ^18^F-FDG PET/CT (three dead at the time of the analysis). 

Among late-NET G3, before the NET G3 diagnosis, six and two patients had discordant and perfectly concordant (100%) functional imaging, respectively. After NET G3 diagnosis, only one patient still showed a complete discordance between two tracers (concordance of 0%), 3/6 showed an improving concordance in 25% of the lesions, while 1/6 reached a complete concordance between ^18^F-FDG PET/CT (negative before) and ^68^Ga-DOTA-TOC PET/CT. 

One of the patients with a 100% concordance before NET G3 diagnosis showed a later discordance due to a decrease in receptorial expression and an ^18^F-FDG uptake prevalence. The patient died just less than 2 mo after NET G3 diagnosis.

Two patients that showed an improving concordance between functional imaging before vs. after NET G3 diagnosis were dead at 39 mo of follow-up; those patients with concordant imaging after NET G3 diagnosis instead were all alive at the time of the analysis.

**Figure 5:**
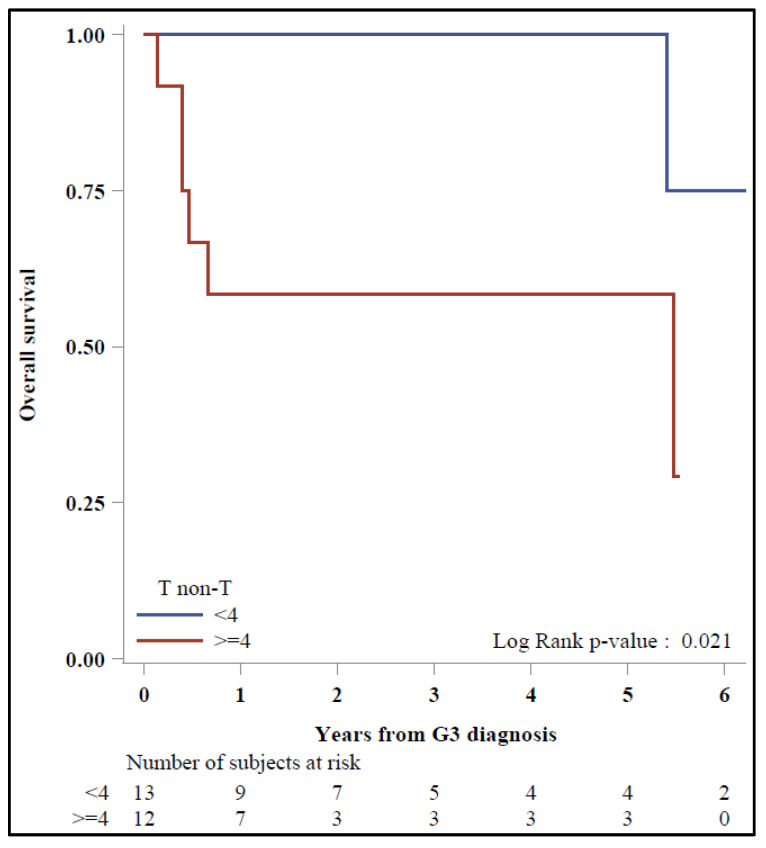
Overall survival by T/non-T ratio.

Finally, among patients with positive ^18^F-FDG PET/CT after NET G3 diagnosis (17/22 upfront and all late-NET G3), in the Kaplan–Meier plots, 5-y OS was 100% vs. 58.3% with a prognosis of 31 vs. 19 mo, respectively, for < vs. ≥4 T/non-T patients (*p* = 0.021) (Figure 5). 

## 4. Discussion

The present retrospective analysis focused on the prognostic role of functional imaging in a NET G3 population managed in the NEN-dedicated MDT of our institute. Since the introduction of the NET G3 definition in GEP NEN classification, we observed different clinical behavior and response to treatments, suggesting the existence of at least different clinical entities within this novel category. In a previous analysis, we identified the possible different clinical, radiological, and pathological classes. In the present analysis, we further investigated the differences in terms of functional imaging and a prognostic role of the concordance between the radiotracers.

In the era of theranostics, the diagnostic and therapeutic application of nuclear medicine, and of the personalization of the therapeutic approach, the need for prognostic and predictive tools enabling a more accurate patient selection for management is rising. Several authors have already reported a prognostic role of ^18^F-FDG and ^68^Ga-DOTA-TOC PET/CT on NENs [9,10,11]. However, most studies on this topic have encompassed NET G3 within the NEC subgroup, leaving the role of functional imaging in this category still unclear. Over the years, the analysis of the ^18^F-FDG and ^68^Ga-DOTA-TOC PET/CT combination has also resulted in a useful tool for NEN characterization and management. Recently, Carideo et al. conducted a systematic review of the prognostic role of combined ^68^Ga-DOTA-TOC and ^18^F-FDG PET /CT in GEP-NENs management. Furthermore, in this in-depth review, only one of eight selected papers specified the number of NET G3 patients but, then, the authors analyzed the subcategory with NET G2 with Ki-67 >10% [12,13].

Regarding the concordance/discordance, Chan et al. proposed the NETPET scoring scheme, a classification system of NENs based on the results of ^18^F-FDG and ^68^Ga-DOTA-TOC PET/CT on the single, initial lesions. The authors identified three prognostic major categories between the P1 class, which encompassed patients with positive ^68^Ga-DOTA-TOC and negative ^18^F-FDG PET/CT, and P5, those with negative ^68^Ga-DOTA-TOC and positive ^18^F-FDG-PET/CT [14]. Additionally, in this study, the number of NET G3 patients was not specified. 

To our knowledge, this is the first study focusing on the prognostic impact of ^18^F-FDG and ^68^Ga-DOTA-TOC PET/CT in the NET G3 category and evaluated possible changes in radiotracer distribution throughout the clinical history. 

First, we analyzed the sample in accordance with the homogeneity/inhomogeneity of functional imaging within the same exam. Several studies have reported a role of ^68^Ga-DOTA-TOC PET/CT SUVmax in stratifying NEN patients into different prognostic categories [10,15]. Nonetheless, this parameter is known to be affected by several biases that may reduce the reliability and reproducibility of the imaging in different settings [16]. 

For these reasons, we decided to classify NET G3 in accordance with the radiotracer distribution, reporting some results that may be more reproducible irrespective of the interpersonal differences and differences in techniques. In the same way, we analyzed the occurrence of further changes in radiotracer distribution during the clinical history of the NET G3 population.

Next, we analyzed the concordance between ^18^F-FDG and ^68^Ga-DOTA-TOC PET/CT based on a percentage and not on the number of lesions: this choice allowed us to analyze all the images with the same method irrespective of the tumor burden. Dividing NET G3 patients into homogeneous/inhomogeneous and concordant/discordant, we reported some interesting findings. A better prognosis for patients with homogeneous ^68^Ga-DOTA-TOC PET/CT and a trend for those with a negative/inhomogeneous ^18^F-FDG PET/CT was demonstrated, in accordance with the literature above-mentioned. 

Among the upfront-NET G3, we reported a significantly higher survival for patients with discordant images and a prevalence of receptorial expression vs. ^18^F-FDG uptake. Interestingly, while the average survival of the former (56.5 mo) may be comparable to that of other larger case series of NET G3 [17], the latter survival suggested a more aggressive behavior (9.5 mo) closer to NEC prognosis. The combination of these, specific clinical and radiological features might aid physicians in further subclassifying NET G3 patients and in distinguishing NENs with a biological behavior closer to NET G2 from the others, closer to NECs. Therefore, besides the prognostic role in NET G3 classification, these factors may offer useful tools for practical and therapeutic implications.

Among the late-NET G3, when analyzing the results of functional imaging during the clinical history, we also found interesting results. First, although no significant differences were reported by comparing all upfront and late-NET G3 patients, the latter presented a better prognosis when compared with the upfront-NET G3 subgroup with discordant imaging and a prevalent ^18^F-FDG uptake (64 vs. 9.5 mo). Second, as already described in the literature, the increase in ^18^F-FDG uptake may worsen the prognosis. However, the results of our analysis suggested that the ^18^F-FDG uptake increase may describe two prognostic subgroups among late-NET G3. On one hand, patients who had a consensual increase in receptorial expression and maintained the concordance between ^18^F-FDG and ^68^Ga-DOTA-TOC PET/CT during the medical history were all alive 64 mo after the NET G3 diagnosis. On the other hand, patients with previous concordant PET/CT experienced an improvement only in metabolic uptake during the medical history. The patients with these characteristics died less than 2 mo after NET G3 diagnosis. Future prospective studies on larger samples and with a longer follow-up may underline the differences between these two subcategories of NET G3. 

Studies on ^18^F-FDG PET/CT have already reported a correlation between Ki-67 level and SUVmax, suggesting a ^18^F-FDG SUVmax prognostic role in high-grade NENs [9,11]. Some authors have also reported a correlation between high SUVmax and a worse response rate to PRRT [18], speculating on a more aggressive systemic treatment to be added to PRRT in NENs with high glycolytic activity [19]. Nevertheless, although the most used parameter in ^18^F-FDG PET/CT is SUVmax, several shortcomings such as uptake time dependence, variability of the arterial input function and the susceptibility to errors in scanner calibration may influence its quantification [20]. Zhang et al. recently reported a prognostic superiority of the T/non-T ratio than ^18^F-FDG SUVmax in the stratification of glioma patients. Therefore, since the acquisitions were performed in different PET scanners or in different clinical situations, we chose to analyze the T/non-T ratio to reduce the interpersonal variability that can affect the SUVmax value [21]. Considering the worse prognostic role of ^18^F-FDG uptake in NENs and the findings above, we explored the role of T/non-T ratio instead of SUVmax in the NET G3 classification strategy. We found a better prognosis for patients with <4 vs. ≥4 T/non-T ratio, which was a prognostic factor independent of the concordance and receptorial/metabolic prevalence. 

Therefore, despite the uniqueness of the pathologically well-defined category of NET G3, we might speculate that (1) upfront subclass with discordant ^18^F-FDG and ^68^Ga-DOTA-TOC PET/CT and (2) prevalent metabolic uptake, (3) experiencing an improvement in metabolic uptake that (4) achieves the ≥4 T/non-T ratio, represents the worst prognosis subcategory than to their opposites. 

While the cases were centralized within a NEN-dedicated MDT, the central pathology specimens and nuclear medicine images reviewed represent the strengths of our analysis, and of course, the retrospective design of the study led to several biases; these, together with the small size (we described only eight late-NET G3 cases, the most with a previous NET G2) and the heterogeneity of the sample represent the greatest limitations of our analysis. Indeed, our study encompasses patients with different stages at diagnosis, for example, two patients showed no evident disease, five patients had received at least one locoregional treatment of the primary, while all the others had a metastatic disease at the time of analysis. Furthermore, patients in our sample had received different systemic therapies in different moments of their medical, which might make it difficult to interpret the outcomes. Indeed, the heterogeneity of the systemic treatments did not allow us to provide data on progression free survival or response rate. Finally, the retrospective design of the study did not allow for homogeneity and systematicity or of histologic sampling, nor for the execution of the functional imaging. Therefore, the sample was heterogeneous for the site and time of sampling and for four patients, the dual-tracer PET/CT was performed over 3 mo of each other. 

Future prospective analyses on the predictive role of these features are needed in order to make a better treatment selection for NET G3 patients.

## 5. Conclusions

Since the introduction of NET G3 into the classification of GEP NENs, differences in terms of prognosis and response to systemic treatments revealed a highly heterogenous disease. Our study suggests the clinical need for a multiparametric definition of NET G3 in whom dual-tracers PET/CT imaging should be performed to stratify patients at higher risk. Accordingly, we designed a prospective observational study on NET G3 (TAPIOCA, IEO 1519) in this specific topic, presently on-going at our institution.

## Figures and Tables

**Table 1:** Features of NET G3 patients.

Variable	Level	NET G3		*p*-Value	Overall (*N* = 30)
		Upfront (*N* = 22)	Late (*N* = 8)		
**Age (y), *N* (%)**	<50	7 (32)	2 (25)	1.00	9 (30)
	50+	15 (68)	6 (75)		21 (70)
	Median (min–max)	55 (34–70)	56 (44–61)	0.85	56 (34–70)
**Sex, *N* (%)**	F	7 (32)	6 (75)	0.049	13 (43)
	M	15 (68)	2 (25)		17 (57)
**Primary site of disease, *N* (%)**	Pancreas	13 (59)	7 (88)	0.85	20 (67)
	Ileum	3 (14)	0 (0)		3 (10)
	Rectum	2 (9)	0 (0)		2 (7)
	Stomach	2 (9)	1 (13)		3 (10)
	Unknown-primary	2 (9)	0 (0)		2 (7)
**Ki-67, *N* (%)**	≤30	10 (45)	6 (75)	0.23	16 (53)
	>30	12 (55)	2 (25)		14 (47)
	Median (min–max)	33 (21–70)	30 (23–45)	0.34	30 (21–70)
**Functioning, *N* (%)**	No	18 (82)	7 (88)	1.00	25 (83)
	Yes	4 (18)	1 (13)		5 (17)
**^68^Ga-DOTA-TOC PET/CT, *N* (%)**	Negative	2 (9)	0 (0)	1.00	2 (7)
	Positive and inhomogeneous	4 (18)	1 (13)		5 (17)
	Positive and homogeneous	16 (73)	7 (88)		23 (77)
**^18^F-FDG-PET/CT, *N* (%)**	Negative	5 (23)	0 (0)	0.47	5 (17)
	Positive and inhomogeneous	7 (32)	4 (50)		11 (37)
	Positive and homogeneous	10 (45)	4 (50)		14 (47)
**Concordance Ga-FDG, *N* (%)**	<75%	16 (73)	5 (63)	0.67	21 (70)
	≥75%	6 (27)	3 (38)		9 (30)
**Prevalence, *N* (%)**	^68^Ga-DOTA-peptide PET/CT	11 (50)	4 (50)	0.76	15 (50)
	^18^FDG-PET/CT	6 (27)	1 (13)		7 (23)
	None	5 (23)	3 (38)		8 (27)
**T non-T, *N* (%)**	<4	9 (53)	4 (50)	1.00	13 (52)
	≥4	8 (47)	4 (50)		12 (48)
	Median (min–max)	4.0 (1.8–8.9)	5.4 (1.6–13.0)	0.68	4.0 (1.6–13.0)

^1^ Among 8 patients with late NET G3, this was the distribution of the years between first diagnosis and G3 diagnosis: Median (min–max): 4.0 (2.3–8.0). ^2^ For “Late NET G3”, values “after” of 68Ga-DOTA-peptide PET/CT, 18FDG-PET/CT, concordance Ga-FDG, prevalence, and T non-T were considered. ^3^ For “Late NET G3”, this was the distribution of “before” and “after” values of T non-T: *“Before”: Median (min-max): 3.0 (2.4–4.0). “After”: Median (min-max): 5.4 (1.6–13.0).*

## Data Availability

IEO shall be classified as autonomous data controllers pursuant to Regulation (EU) 2016/679 of the European Parliament and Council of 27 April 2016 (GDPR).
